# The Pomegranate: Effects on Bacteria and Viruses That Influence Human Health

**DOI:** 10.1155/2013/606212

**Published:** 2013-05-20

**Authors:** Amy B. Howell, Doris H. D'Souza

**Affiliations:** ^1^Marucci Center for Blueberry and Cranberry Research, Rutgers, The State University of New Jersey, Chatsworth, NJ 08019, USA; ^2^Department of Food Science and Technology, The University of Tennessee, Knoxville, TN 37996, USA

## Abstract

Pomegranates have been known for hundreds of years for their multiple health benefits, including antimicrobial activity. The recent surge in multidrug-resistant bacteria and the possibility of widespread global virus pandemics necessitate the need for additional preventative and therapeutic options to conventional drugs. Research indicates that pomegranates and their extracts may serve as natural alternatives due to their potency against a wide range of bacterial and viral pathogens. Nearly every part of the pomegranate plant has been tested for antimicrobial activities, including the fruit juice, peel, arils, flowers, and bark. Many studies have utilized pomegranate peel with success. There are various phytochemical compounds in pomegranate that have demonstrated antimicrobial activity, but most of the studies have found that ellagic acid and larger hydrolyzable tannins, such as punicalagin, have the highest activities. In some cases the combination of the pomegranate constituents offers the most benefit. The positive clinical results on pomegranate and suppression of oral bacteria are intriguing and worthy of further study. Much of the evidence for pomegranates' antibacterial and antiviral activities against foodborne pathogens and other infectious disease organisms comes from *in vitro* cell-based assays, necessitating further confirmation of *in vivo* efficacy through human clinical trials.

## 1. Introduction: Pomegranates and Their Effects on Human Bacteria

**Figure 1:**
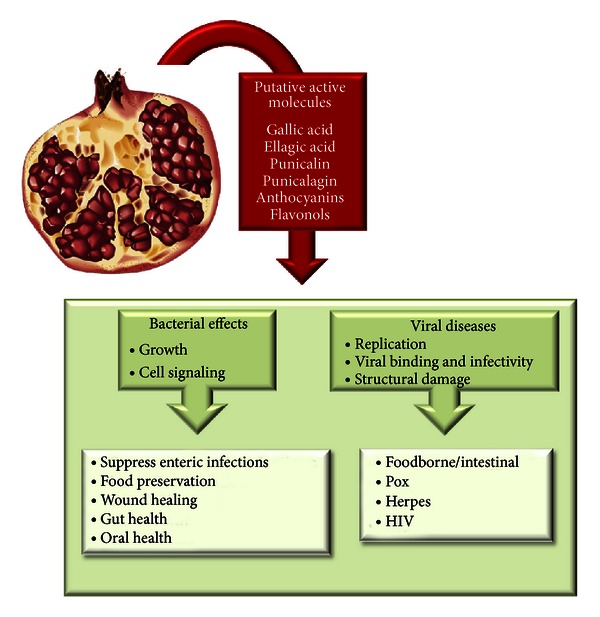


Pomegranates (*Punica granatum* L.) have a long history of antibacterial use dating back to biblical times. Egyptians used pomegranates to treat a number of different infections [1]. It was utilized as a traditional remedy for thousands of years under the Ayurvedic system of medicine, with extracts from the rind of the fruit and bark of the tree being effective against diarrhea and dysentery [2]. Over the years there have been many small studies undertaken in different areas of the world on the bactericidal effects of pomegranates on a number of highly pathogenic and drug-resistant strains. These studies normally determine bactericidal potency of different extracts of the pomegranate plant against a range of different bacteria, utilizing disc diffusion assays or minimum inhibitory concentration (MIC). Methanol extracts of the fruit, especially the peel, exhibit the broadest antibacterial activity [3–8] (Table 1), which can vary depending on the pomegranate variety tested [9]. Methanol extracts of pomegranate are high in hydrolyzable tannins (punicalins and punicalagins), ellagic acid, a component of ellagitannins, and gallic acid, a component of gallotannins [10] (Figure 1). Mass spectrometry data shows that pomegranate contains oligomeric ellagitannin with a degree of polymerization of up to 5 core glucose units [11]. These molecules may be the most potent antibacterial compounds in pomegranate. However, other compounds also have activity and may contribute synergistically as mixtures to bring about the effects, including anthocyanins (pelargonidin-3-galactose and cyanidin-3-glucose) and flavonols (quercetin and myricetin) [8]. 

## 2. Evidence for Pomegranate in Controlling Bacteria That Affect the Human Body

### 2.1. Effects on Enteric Bacteria

Enteric bacteria can be either probiotic and exert beneficial effects on the gut microflora, or they can be pathogenic and cause life-threatening infections and disease. Pomegranate has positive effects on both probiotic and pathogenic bacteria. It also shows promise in food preservation by protecting against pathogenic bacteria that can cause food poisoning.

#### 2.1.1. Inhibition of Enteric Pathogenic Bacteria

Many foodborne bacteria cause serious gastrointestinal infections, such as enterohemorrhagic *Escherichia coli *(*E. coli*) O157:H7 which can lead to hemorrhagic diarrhea. These infections can be life-threatening to young children and the elderly. There is an incentive to find alternative control measures, such as plant and herbal extracts, especially in lesser-developed countries where traditional antibiotics may not be readily available. In Thailand, a study was undertaken in which extracts of pomegranate were tested for their antibacterial activity against different strains of *E. coli*, including 3 strains of *E. coli* O157:H7 [12]. Growth inhibition zones, using the agar disc diffusion method, ranged from 7 to 17 mm. An aqueous extract of pomegranate was highly effective against *E. coli* O157:H7 with MIC and minimal bactericidal concentration (MBC) values of 0.19 and 0.39 mg/mL, respectively. In another Thai study, an ethanolic extract of pomegranate had MICs of 0.49 to 1.95 mg/mL and MBCs of 1.95 to 3.91 mg/mL against *E. coli* O157:H7 [13]. This extract exhibited both bacteriostatic and bactericidal activities, indicating that it may be an effective adjunct treatment for *E. coli* O157:H7 infection. 

Pomegranate has exhibited bactericidal activity against other food and waterborne pathogenic bacteria including *Salmonella* Typhi (*S*. Typhi)[14, 15], *Vibrio cholerae* [16, 17], *Yersinia enterocolitica* [7], *Shigella* spp. [16, 18], and *Listeria monocytogenes* (*L. monocytogenes*) [7, 19, 20]. Typhoid fever (causal agent, *S*. Typhi) is a life-threatening enteric infection that can be transmitted by consuming food or drinking water contaminated with feces from an infected person. It is more common in less industrialized countries. Extracts of pomegranate fruit pericarp were tested by agar well diffusion and found to be highly active when compared to a reference concentration-response curve for ampicillin [14]. In another study which screened plants of importance in the Ayurvedic system of medicine, strong antibacterial activity was exhibited by the methanol extracts of pomegranate [15]. *V. cholerae*, the cause of cholera infections, is most commonly acquired from feces-infected drinking water. In one study examining bactericidal activity of plants used by Peruvian people to combat cholera, pomegranate peel extract and tea infusions were effective [17]. *Shigella* spp. are an important cause of diarrhea and dysentery in the Mexican population. In one study, ethanolic pomegranate extracts exhibited greater antibacterial activity than the antibiotic chloramphenicol, but lower activity than trimethoprim [18]. *Shigella sonnei* showed the highest susceptibility to the extracts.

Pomegranate extract was evaluated in several studies for the ability to decontaminate meat surfaces and maintain food quality. A pomegranate peel extract at 250 *μ*g/mL was most effective at inhibiting antibiotic resistant strains of *Salmonella* Typhimurium (*S*. Typhimurium) and *Staphylococcus aureus *(*S. aureus*) on meat surfaces and improved sensory evaluations of quality [21]. In another study, dipping raw chicken breasts in 0.02% pomegranate fruit juice solution reduced protein oxidation, inhibited microbial growth, and increased sensory acceptability for up to 12 days of refrigerated storage at 4°C [22]. Dried pomegranate juice powder was heated to 100 degrees C for 0, 30, 60, or 120 minutes and added at 2% (wt/wt) to ground top round beef to determine if heat would alter the antilisterial activity of the powder [20]. The meat was then cooked and inoculated with individual *L. monocytogenes* strains. Samples of meat stored at 5°C were taken at days 1, 8, 14, and 21 and plated on media for evaluation of bacterial growth. All the heat-treated pomegranate juice powder treatments significantly inhibited growth of all five *L. monocytogenes* strains in refrigerated ground cooked beef by 1.80 to 4.61 log CFU/g at day 21, suggesting that heating does not impact the antilisterial activity of pomegranate. In another study, an 80% methanolic pomegranate peel extract resulted in a >1 log(10) reduction of *L. monocytogenes* in fish during storage at 4°C [7]. One study examined the effectiveness of pomegranate peel to inhibit growth of *L. monocytogenes*, *S. aureus*, and *Salmonella enterica* in cheese at room temperature (~23°C) [19]. The pomegranate treatment increased the stability of cheese against lipid oxidation, improving shelf life. A pomegranate sour sauce had an antimicrobial effect when mixed with lettuce, spring onion, and parsley, either inoculated with *S. aureus* and *E. coli* O157:H7 or containing the naturally existing bacterial flora [23].

#### 2.1.2. Beneficial Effects on Enteric Probiotic Bacteria

Preservation and/or enhancement of probiotic bacteria in the gut is important for maintaining gastrointestinal health. A hydrolyzable tannin-rich pomegranate by-product (POMx) incubated with faecal bacteria resulted in formation of the dibenzopyranone-type urolithins which enhanced the growth of *Bifidobacterium *spp. and *Lactobacillus* spp. [24]. In another study, POMx significantly enhanced the growth of *Bifidobacterium breve* and *Bifidobacterium infantis* while inhibiting the growth of pathogenic clostridia and *Staphylooccus aureus *[25]. The effects of individual pomegranate ellagitannins were evident but less pronounced than the tannin mixture in the POMx formulation. 

Pomegranate extract had a beneficial effect on rumen bacterial populations in lactating cows [26]. The peel extract was fed at levels of 1, 2, or 4% on voluntary intake. The supplementation had a significant positive dose-dependent effect on the entire ruminal bacterial community, as determined by automated ribosomal intergenic spacer analysis. In cows fed at the 4% extract level, there were significant increases in digestibility of dry matter, crude protein, and neutral detergent fiber, as well as milk yields.

### 2.2. Enhancement of Wound Healing

Pomegranate skin preparations hold promise in increasing the rate of wound healing. Pomegranate peel (5% methanolic extract) prepared as an ointment was applied to guinea pig wounds daily for 12 days [27]. The treatment significantly enhanced wound healing by increasing collagen, DNA and protein synthesis as well as contraction rate and tensile strength. The extract exhibited significant antibacterial activity against wound bacteria, including strains of *Pseudomonas aeruginosa*, *S. aureus*, *E. coli*, *Klebsiella pneumoniae *(*K. pneumoniae*), *Salmonella* Anatum, *S*. Typhimurium, and *Streptococcus pneumoniae*. There were no toxic effects noted from use of the skin ointment. Another study using the methanolic extract of pomegranate peels formulated into a 10% (wt/wt) water-soluble gel showed similar enhancements in wound healing in a Wistar rat model compared to a commercial topical antibacterial product [44]. The group treated with 5.0% gel had a 59.5% increase in contraction of the skin, a 2-fold increase in collagen content, and positive microscopic changes to the skin. Participant's wounds were completely healed after 10 days, compared to 16–18 days for the group receiving a blank control gel. The activity was postulated to be due to the high phenolic content (44%) in the peel extract. 

Pomegranate flower extracts also hold promise for augmenting wound healing. A diethyl ether flower extract was applied to wounds in alloxan-induced diabetic rats at a dose of 200 mg/kg/day [28]. The extract-treated diabetic rats showed significant reduction in the wound area when compared with the control. Another study in which flower extracts of pomegranate were applied to wounds resulted in decreased wound size compared to the control group and a significant increase in the rate of wound contraction and collagen turnover [29].

### 2.3. Reductions in Oral Bacteria

Studies suggest a role for pomegranate extracts in reducing and preventing pathogenic dental bacteria and reducing the risk of plaque, gingivitis, and periodontal disease. Many of these studies are human clinical trials. 

#### 2.3.1. *In Vitro* Antibacterial Activity against Oral Bacteria

The effects of three different concentrations of a methanolic pomegranate peel extract at 4 mg/mL, 8 mg/mL, and 12 mg/mL on growth of dental bacteria were compared using the disc diffusion method [30]. All concentrations of the pomegranate extract had antibacterial activity against *S. aureus* and *S. epidermidis*. Extract concentrations of 8 mg/mL and 12 mg/mL were effective against *L. acidophilus*, *S. mutans*, and *S. salivarius*. The extract did not inhibit *Actinomyces viscosus*. In another similar *in vitro* study, ethanol and water extracts of pomegranate both had inhibitory effects against *S. mutans* and *Porphyromonas gingivalis* (*P. gingivalis*) [32]. A Brazilian *in vitro* study investigated the antimicrobial effect of a pomegranate-based oral gel (made from an extract of dried peel combined with Carbopol, water, and triethanolamine) against *Streptococcus sanguis*,* Streptococcus mitis*, and *S. mutans* [31]. The MICs required to inhibit adherence of the bacteria to glass were assessed using increasing and doubled concentrations of the diluted solution of the pomegranate gel at concentrations ranging from 1 : 1 to 1 : 1024. The MICs of adherence of pomegranate gel against the bacteria were 1 : 16 for *S. mutans* and *S. sanguis* and 1 : 128 for *S. mitis*. These results suggest that pomegranate gel might be useful in the control of adherence of different bacteria in the oral cavity. In other *in vitro* studies, pomegranate extract also inhibited strains of periodontal bacteria, *Aggregatibacter actinomycetemcomitans*, *P. gingivalis*, *Prevotella intermedia* [33], *Klebsiella*, *E. coli*, and *Proteus* spp. [34]. 

#### 2.3.2. Clinical Studies on Prevention of Dental Plaque

Several clinical trials have explored the effectiveness of pomegranate extract rinses on reductions in oral plaque [33, 34]. In one trial, the amount of plaque accumulation was measured at days 0 and 5 in thirty periodontally healthy volunteers who refrained from all mechanical oral hygiene measures for 4 days and instead used either pomegranate extract, chlorhexidine, or a placebo rinse twice daily [33]. At day 5, those volunteers using the pomegranate extract had significantly less plaque buildup (*P* < 0.05) than those using the placebo rinse. The pomegranate extract prevented as much plaque as the chlorhexidine rinse. These results on pomegranate's effect on plaque reduction are supported by another human trial in which pomegranate extract rinse was compared to chlorhexidine and placebo rinse [34]. After 24 hours without tooth brushing, plaque samples were taken from sixty healthy, younger patients between the ages of 9 and 25 who wore orthodontic appliances. Dental plaque samples were plated on media for 48 hours, and the number of colony forming units per milliliter (CFU/mL) showed that the pomegranate extract rinse was effective against dental plaque microorganisms, decreasing the CFU/mL by 84%, similar to chlorhexidine (79% inhibition), and significantly different from the control rinse (11% inhibition). Authors speculated that the ellagitannin and punicalagin may be responsible for the antibacterial activity of the pomegranate extract rinse. 

#### 2.3.3. Clinical Studies on Gingivitis

Gingivitis is an inflammation of the gums in response to bacterial plaque biofilms adhering to tooth surfaces. If left untreated, gingivitis may progress to periodontal disease and subsequent tooth loss. There is an incentive to use alternative plant-based preparations as an adjunctive to mechanical therapy in the prevention and treatment of gingivitis, due to the health risks imposed by long-term use of chemical and pharmaceutical preparations and the lack of available dental care in lesser-developed countries. Results of a randomized clinical study of 40 patients with chronic gingivitis showed that significant improvements were obtained in the group that used a pomegranate extract gel along with mechanical debridement for 7 days when compared with patients using only control gel or mechanical debridement for the 7-day test period [45]. Another placebo-controlled human clinical trial of 32 young adults examined salivary measures relevant to oral health and gingivitis after using a pomegranate extract mouth rinse three times per day for 4 weeks or a placebo rinse [46]. Compared to the control group, those participants using the pomegranate rinse had reduced total protein associated with presence of plaque-forming bacteria, reduced activities related to cell injury, reduced levels of the sucrose-degrading enzyme alpha-glucosidase, and increased activity of the enzyme ceruloplasmin, which protects against oral oxidative stress. Based on these results, the authors suggest the possibility of using pomegranate extracts in oral health products such as toothpaste and mouthwashes.

#### 2.3.4. Clinical Study on Periodontitis

A clinical study was undertaken to test pomegranate peel extract impregnated into biodegradable chips for use subgingivally as an adjunct to scaling and root planning for maintenance of periodontal disease [47]. The pomegranate chips or placebo chips were implanted in 20 patients with gum pocket depths of 5–8 mm. Level of bacterial attachment, bleeding, and gingival and plaque indexes were initially measured and again at 3 and 6 months. After 3 months, the pomegranate treatments resulted in decreased plaque and significant decreases in pocket depth and bacterial attachment compared to placebo. A marker of inflammation (IL-1beta) was also lower at 3 and 6 months compared to baseline. 

### 2.4. Inhibition of Antibiotic-Resistant Bacteria

Methicillin-resistant *Staphylococcus aureus* (MRSA) is any strain of *S. aureus* that has become resistant to beta-lactam antibiotics, including the penicillins and the cephalosporins. These MRSA strains are not necessarily more virulent than antibiotic-susceptible strains of *S. aureus*, but they are more dangerous because they do not respond to first-line antibiotics. MRSA can cause life-threatening infections in people with weakened immune systems, especially in lesser-developed countries where antibiotics are not readily available, and in hospitals and nursing homes. There is evidence from a number of *in vitro* experiments that pomegranate extracts moderately to strongly inhibit cultured MRSA strains [35–41]. In one study, ethanolic extracts of pomegranate were effective at inhibiting 35 hospital isolates of MRSA at MICs of 0.2–0.4 mg/mL [36]. In another study, an extract of high-tannin pomegranate polyphenols at 1 and 5 mg/mL was found to cause 1.1–2.3 log_10_ CFU/mL reduction of the two MRSA strains after 2 hours at 37°C, and to undetectable levels in most strains within 24 hours [39]. Scanning electron microscopy of the bacteria showed that the pomegranate extract caused alterations in the bacterial cell walls after 2 hours of treatment. 

Multidrug-resistant *Acinetobacter baumannii* (*A. baumannii*) is considered to be one of the most difficult bacterial infections to treat. *A. baumannii* survives for prolonged periods under a wide range of environmental conditions and causes serious infection outbreaks in hospitals. It is very difficult to control due to antibiotic resistance, so the need for alternative approaches is under investigation. Pomegranate extract was tested as a resistance-modifying agent of the antibiotic novobiocin against *A. baumannii* using a growth inhibition assay [42]. Pomegranate extract at 250 *μ*g/mL significantly enhanced the antibacterial activity of novobiocin at 1 *μ*g/mL (1/8 × MIC) against *A. baumannii*. 

*Helicobacter pylori* (*H. pylori*) is the causal agent of stomach ulcers, which if left untreated can lead to stomach cancer. However, the bacteria are becoming resistant to the antibiotics used to treat ulcers, making this condition difficult to cure. Disc diffusion was utilized to test the *in vitro* susceptibility of *H. pylori* isolated from patients with gastroduodenal complications to a pomegranate methanol extract [43]. The pomegranate extract exhibited strong activity against *H. pylori* with a mean inhibition zone diameter of 39 mm at 100 *μ*g disc^−1^. Pomegranate peel extracts from nine Iranian cultivars were further assayed against the *H. pylori *isolates. The results demonstrated that most all cultivars showed significant *in vitro* anti-*H. pylori *activity with the mean inhibition zone diameter ranging from 16 to 40 mm at 50 *μ*g disc^−1^.

#### 2.4.1. Enhancement of Antibiotic Susceptibility

Pomegranate enhanced the activity of antibiotics against MRSA in one study [48]. Synergistic activity between a methanolic pomegranate extract and the antibiotics chloramphenicol, gentamicin, ampicillin, tetracycline, and oxacillin ranged from 38% to 73% against clinical isolates of MRSA. The bactericidal activity of the pomegranate extract (0.1 × MIC) with ampicillin (0.5 × MIC) was assessed and determined to be synergistic. This combination increased the lag time to bacterial growth by three hours over that of ampicillin alone and resulted in a 99.9% reduction in MRSA. The mechanism of action of the pomegranate extract was to either inhibit the MRSA NorA efflux pump or to enhance the influx of the antibiotic. 

The antibiotic activity of ciprofloxacin was enhanced by a methanolic pomegranate peel extract against resistant strains of extended-spectrum beta-lactamase-producing *E. coli*, *K. pneumoniae*, and metallo-beta-lactamase-producing *P. aeruginosa* [49]. Synergy with ciprofloxacin was observed in 19 of 49 strains (FIC of 0.125–0.5 for ciprofloxacin) possibly due to the bacterial efflux pump inhibitor activity of the pomegranate tannins. 

### 2.5. Influence on Bacterial Quorum-Sensing Mechanism

Quorum sensing is an intercellular signaling mechanism used by bacteria to communicate as a colony about critical survival issues such as availability of nutrients, defense against other microorganisms, virulence, and biofilm formation. Bacteria produce and detect signaling molecules important for pathogenic bacteria during infection of a host to coordinate their virulence in order to escape the immune response of the host and establish a successful infection. Interference of quorum-sensing signals is a strategy for disease control. Pomegranate inhibited quorum-sensing signals in two bacterial strains, interfering with purple pigment production and bacterial swarming motility in *Chromobacterium violaceum* and *P. aeruginosa*, respectively [50, 51]. The inhibition of these particular processes may be due to direct or indirect interference on quorum-sensing by pomegranate polyphenols or the interactive effect of different compounds present in the extract. 

## 3. Introduction: Pomegranates and Their Effects on Human Viruses

Limited studies have been conducted on the antiviral activities associated with pomegranate and its extracts. The fruit's antiviral effects have been reported against clinically relevant influenza virus, herpes virus, poxviruses, and human immunodeficiency (HIV-1) virus [52–54]. The hydrolyzable tannins and anthocyanins are the main compounds associated with the beneficial effects of pomegranate consumption on other health effects “including antibacterial” [55], and may be responsible for the antiviral activity. In one study, among four flavonoid compounds associated with pomegranates (ellagic acid, caffeic acid, luteolin, and punicalagin), only punicalagin was shown to have inhibitory effects on influenza virus [56]. Natural antimicrobials from plant extracts have become increasingly popular for use as alternative antivirals [57–60]. The increased research and need for such alternatives are based on the many advantages of natural plant antimicrobials. These include the absence of reported/observed toxic effects at recommended doses along with additional benefits such as antioxidant, anticancer, anti-inflammatory, and antimicrobial properties [3, 10, 39, 52, 53, 56, 61–64]. It is possible that pomegranate juice and extracts could be potentially useful in inhibiting viruses transmitted via infected food products, bodily fluids, and so forth. 

## 4. Evidence for Pomegranate in Controlling Viruses That Affect the Human Body

### 4.1. Inhibition of Nonenveloped Clinically Relevant Foodborne Viral Pathogens

Recent studies indicate that viruses cause an estimated 59% of all foodborne illnesses (5.5 million), 27% of hospitalizations, and 12% of deaths in the United States alone [65]. Foodborne virus outbreaks are gaining immense attention due to their increased incidence and scope of illness. 

The epidemiologically significant foodborne viruses include human noroviruses, hepatitis A virus, rotaviruses, Aichi virus, hepatitis E virus, astroviruses, adenoviruses, parvoviruses, and other human enteroviruses and small round structured viruses [66]. Among the foodborne viruses, human noroviruses are the main cause of viral gastroenteritis outbreaks worldwide [65, 67, 68]. Human noroviruses (HNoVs) belong to the family of *Caliciviridae*, being nonenveloped and round in shape with a diameter of 27 to 40 nm. There are currently five genogroups based on nucleic acid sequence analysis, of which primarily genogroup I and frequently genogroups II and IV are associated with human norovirus outbreaks. Earlier studies indicated that HNoVs are estimated to be solely responsible for up to 2.3 million infections, 50,000 hospitalizations, and 300 deaths per year in the US alone [69]. 

As HNoVs cannot be grown in cell culture, cultivable surrogates such as feline calicivirus (FCV-F9) [70], bacteriophage MS2 [71], and murine norovirus (MNV-1) [72] are used in infectivity assays to study antiviral or inactivation effects, though the more recently cultivable Tulane virus is also being researched [73]. HNoVs can be transmitted from person to person, through food, aerosols, water, and contact with fomites [74]. They are reported to have a low infectious dose of 10 to 100 viral particles with symptoms including nausea, vomiting, diarrhea, abdominal pain, and low-grade fever. The infection is self-limiting in healthy individuals and lasts for up to 72 hours. However, newly emergent strains (such as genogroup II.4) have become highly virulent and life-threatening especially to the elderly and immunocompromised [68]. Currently, there are no known vaccines available to prevent norovirus infection or disease onset. In addition, there is no effective treatment option available besides rehydration therapy.

The effectiveness of natural remedies such as pomegranate juice and extracts as alternatives for the treatment or prevention of foodborne norovirus infections needs to be explored more aggressively. Recently, pomegranate juice and polyphenols were shown to have significant antiviral effects against foodborne viral surrogates, FCV-F9, MNV-1, and bacteriophage MS2 [75]. These researchers showed that the juice could decrease low titers (~5 log_10_ PFU/mL) of FCV-F9, MNV-1, and MS2 by 2.56, 1.32, and 0.32 log_10_ PFU/mL, respectively, after 1 hour at room temperature and high titers (~7 log_10_ PFU/mL) by 1.20, 0.06, and 0.63 log_10_ PFU/mL. The most potent effect was on FCV-F9 after treatment with 8, 16, or 32 mg/mL of pomegranate polyphenol (extracted from fresh pomegranate fruit-POM obtained from POM Wonderful). After 1 hour at room temperature, low and high titers of FCV-F9 were completely undetectable. Following 1-hour incubation with 4, 8, or 16 mg/mL pomegranate polyphenol, low initial titers of MNV-1 were reduced by 1.30, 2.11, and 3.61 log_10_ PFU/mL and high initial titers by 1.56, 1.48, and 1.54 log_10_ PFU/mL, respectively. The titer reduction effect of pomegranate against MNV-1 is notable in that this surrogate is considered to be more appropriate than FCV-F9 by some researchers. Bacteriophage MS2 at low initial titers was reduced by 0.41, 0.45, and 0.93 log_10_ PFU/mL and at high initial titers by 0.32, 0.41, and 0.72 log_10_ PFU/mL after incubation with 4, 8, or 16 mg/mL of pomegranate polyphenol, respectively. Overall effectiveness of pomegranate on reduction of virus titer rankings was FCV-F9 > MNV-1 > MS2 for the tested low-titer viruses (5 log_10_ PFU/mL), while for the high-titer viruses (7 log_10_ PFU/mL), the effectiveness rankings were FCV-F9 > MS2 > MNV-1 [75]. Pomegranate juice contains total phenolics of 3.6 mg/mL [55]; therefore, pomegranate polyphenols were tested at a similar concentration (4 mg/mL) and exhibited consistently greater antiviral effects than that of pomegranate juice against all viruses at both high and low titers [75]. This difference may be due to variability in composition and bioavailability of polyphenols in juice versus those extracted in pure form. In addition, the antiviral effects are not pH-dependent, as no differences in bioactivity were noted when juice pH was changed from 3.4 to 7.0. Given that MNV-1 is quite resistant to most treatment conditions, including pH and heat [73], but inhibited by pomegranate juice and its polyphenols, it is possible that additional research will reveal a role for pomegranate as a natural alternative for treating and/or preventing human norovirus infections. 

Comparison of the effects of cranberry juice, grape juice, and orange juice on the infectivity of foodborne viral surrogates revealed that the titer reduction for FCV-F9, MNV-1, and MS2 followed the order of cranberry juice > pomegranate juice > grape juice > orange juice in general, with the exception that grape juice had a greater effect on high-titer FCV-F9 than cranberry juice [75].

When the time-dependent effects of pomegranate juice and polyphenols at 2 and 4 mg/mL against foodborne viral surrogates were further studied, varied titer reduction rates of FCV-F9, MNV-1, and MS2 over 1 hour at room temperature were obtained [59]. There were no significant differences in titer reductions when comparing different brands of commercial pomegranate juice; however, titer reduction levels were affected by different storage times. For all three viruses, ≥50% of the total reduction was found to be achieved within 20 minutes. Interestingly, upon immediate mixing with pomegranate juice or 2 or 4 mg/mL pomegranate polyphenols, FCV-F9 was reduced by 1.35, 1.97, and 2.39 log_10_ PFU/mL, respectively, with further reductions of 1.74, 2.02, and 2.68 log_10_ PFU/mL within the next 20 minutes. Compared to FCV-F9, bacteriophage MS2 and MNV-1 titers were not significantly reduced using this experimental regime. These results indicate an *in vitro* effect of pomegranate against human norovirus surrogates; however, further *in vivo* work is necessary to determine if clinically relevant therapeutic or preventive uses are viable.

Konowalchuk and Speirs [76] found that <1% of 3 log PFU poliovirus/0.05 mL survived after storage at 4°C for 24 hours in pomegranate juice, though the mechanism of action was unknown. Poliovirus is transmitted through the fecal-oral route in a manner similar to other enteroviruses, being a nonenveloped RNA virus. As preventive measures, poliovirus vaccines are available.

### 4.2. Mechanism of Action against Human Norovirus Surrogates

In order to understand the mechanism of action, the host cell monolayers for the respective viruses were treated with pomegranate juice and polyphenols prior to or after infection, where reduced infectivity of FCV-F9 and MNV-1 was obtained [75]. Greater effects in titer reduction were observed when the treatment was performed prior to infection (corresponding to attachment/adsorption stage) than after infection (corresponding to replication stage), suggesting that pomegranate juice and its polyphenols may play a role in preventing virus binding to the host cell receptors by blocking the cell surface receptors or the virus surface ligands. It was postulated that further work using transmission electron microscopy may determine if these polyphenols cause structural damage to the virus or virus capsid. 

Recently, a cranberry-pomegranate juice blend was shown to reduce the specific binding ability of human NoV P particles to salivary human histoblood group antigens (HBGAs) using an enzyme-linked immunosorbent assay (ELISA), where the binding pattern is reported to correspond with the probability of infection [77]. HBGAs are complex carbohydrates present on red blood cell surfaces, the mucosal epithelium of respiratory, genitourinary, and digestive tracts and as free oligosaccharides in saliva, intestinal contents, milk, and blood. These researchers reported that cranberry juice at concentrations of 10 and 100% and cranberry-pomegranate at concentrations of 1% to 100% were found to reduce the binding of human NoV strains specifically to certain types of human HBGAs, in agreement with the study by Su et al. [75] that used infectivity plaque assays. Li et al. [77] also postulated that the interaction of plant polyphenolic compounds with the viral capsid protein may cause irreversible damage or reversible blocking of certain regions/areas of the capsid protein. 

### 4.3. Inhibition of Other Clinically Relevant Viruses

Pomegranate extracts have also shown antiviral effects against influenza virus, HIV-1 and poxviruses [52, 53, 76]. Influenza virus continues to be a major cause of morbidity and mortality each year with 31,000 deaths reported yearly in the US, despite access to vaccines [52]. However, frequent recombination events and viral evolution necessitate the change in vaccine composition requiring administration of new vaccines yearly. Researchers have shown that pomegranate polyphenols were virucidal against influenza A virus, suppressed the replication of the virus in host cells, and inhibited agglutination of chicken red blood cells caused by the virus using real-time polymerase chain reaction, a plaque assay, and a median tissue culture infective dose 50% hemagglutination assay [52]. They also showed that among four polyphenols (ellagic acid, caffeic acid, luteolin, and punicalagin), punicalagin was found to be the most effective anti-influenza component, blocking replication of influenza virus RNA and inhibiting agglutination of chicken red blood cells by the virus. 

Sundararajan et al. [78] also showed that the acidity of pomegranate juice and concentrated liquid pomegranate extract (POMxl) solutions contributed to rapid anti-influenza activity, whereas pomegranate polyphenol (PP) powder (POMxp) did not. A 5-minute treatment at room temperature with 800 *μ*g/mL PP was shown to result in at least a 3 log titer reduction of influenza viruses PR8 (H1N1), X31 (H3N2), and a reassortant H5N1 virus derived from a human isolate. Loss of hemagglutinating activity was reported to accompany the loss of influenza infectivity, with decreased antibody binding to viral surface molecules after treatments with PP. Viral structural damage was also reported using electron microscopic analysis of PP-treated viral particles. However, they found that the antiviral activity was less against avian isolates of one coronavirus and reassortant H5N1 influenza viruses. Kotwal [54] suggested that pomegranate juice can neutralize the infectivity of diverse enveloped viruses and a number of subtypes of a given enveloped virus, indicating potential for development as a treatment option that can be broadly effective against pandemic viruses like HIV, potentially pandemic viruses like influenza, and some carcinogenic viruses. It was shown that influenza A/HK/x31(H3N2), influenza A/Vietnam/1203/04 (H5N1), and a reassortant x31 containing the NS gene segment of an H5N1 isolate were inactivated when treated for 5 minutes at 37°C with pomegranate juice [54].

In 2003, the AIDS pandemic was reported to claim 30 million lives that resulted in ~14,000 new HIV-1 global infections daily [53]. In the absence of vaccines, antiretroviral chemotherapeutics have been used to decrease HIV-1 symptoms mainly in developed countries. Neurath et al. [53] showed that HIV-1 entry inhibitors from pomegranate juice are adsorbed onto corn starch, block HIV-1 binding to CD4 and CXCR4/CCR5 host cell receptors, and inhibit infection by primary virus clades A to G and group O. These researchers showed the potential of producing anti-HIV-1 microbicides from naturally safe food sources. 

### 4.4. Mechanisms of Action of Other Polyphenols

Several other mechanisms of antiviral activity have been proposed for nonpomegranate polyphenols, which could offer valuable insights for researchers studying the antiviral mechanisms related to pomegranate consumption. Haslam [79] suggested that plant polyphenols exert a direct action on the viral particles, inhibiting the adsorption of the virus to the host cell receptors. One study found that proanthocyanidin A-1 inhibited viral attachment and penetration and affected the late stages of herpes simplex virus type 2 infection [80]. Liu et al. [81] determined that the inhibitory effect of tea polyphenols is through multiple mechanisms of action, including inhibiting HIV-1 reverse transcriptase and protease activity, blocking gp120-CD4 interaction by binding to cellular CD4 molecules, and destroying viral particles. Further studies are needed to identify additional antiviral mechanisms of action associated with pomegranate and its constituents. 

## 5. Conclusions

There are a number of studies on pomegranate and their antimicrobial activities against bacteria and viruses, with mechanisms of actions including pH-independent bacterial and viral growth inhibition, effects on bacterial cell signaling, reductions in viral infectivity and binding to host cell receptors, and structural damage to viruses. However, many of the study results are based on *in vitro* and cell-based assays. Therefore, the applicability of the results to human health is mainly focused on diseases and infections that occur topically, such as those in the oral cavity or on the skin surface. It is difficult to extrapolate *in vivo *effects on infection from *in vitro* results using unmetabolized pomegranate juice or compounds on microbes. In addition, it is difficult to compare results of studies that do not standardize the treatment extracts for the active components. Standard procedures should be adopted by the pomegranate industry that utilize the same quantification methods. A method for determining ellagitannin levels using a novel pomegranate standard has been published, which could be helpful in addressing this issue [82, 83]. The current studies do support potential benefit of pomegranate extracts in food preservation and decontamination. This application could be particularly useful in lesser-developed countries where food sanitation can easily be compromised. Results of the studies on antibacterial benefits of pomegranate extracts against dental bacteria and infections hold promise because they are clinically-based. Additional larger-scale trials should be conducted to confirm the benefits. 

## Figures and Tables

**Table 1:** Effect of pomegranate extracts on growth of bacteria that influence human health.

Bacteria	Pomegranate extract	Growth inhibition (−) or promotion (+)	Citation
Enteric			
*Escherichia coli* O157:H7	Peel, bark	−	[2, 12]
*Salmonella* Typhi	Peel	−	[14, 15]
*Salmonella* Typhimurium	Peel	−	[21]
*Salmonella enterica serovars *	Peel		[19]
*Vibrio cholerae *	Peel	−	[16, 17]
*Yersinia enterocolitica *	Peel	−	[7]
*Shigella* spp.	Peel	−	[16, 18]
*Shigella sonnei *	Peel	−	[21]
*Listeria monocytogenes *	Peel, dried juice powder	−	[7, 19, 20]
*Staphylococcus aureus *	Peel, juice, and POMx	−	[19, 21, 22, 25]
*Clostridium* spp.	POMx		
Probiotic			
*Bifidobacterium *spp.	POMx	+	[24]
*Lactobacillus* spp.	POMx	+	[24]
*Bifidobacterium breve *	POMx	+	[25]
*Bifidobacterium infantis *	POMx	+	[25]
Wound			
*Pseudomonas aeruginosa *	Peel, flower extract	−	[27–29]
*Staphylococcus aureus *	Peel	−	[27–29]
*Escherichia coli *	Peel	−	[27–29]
*Klebsiella pneumoniae *	Peel	−	[27–29]
*Salmonella* Anatum	Peel	−	[27–29]
*Salmonella* Typhimurium	Peel	−	[27–29]
*Streptococcus pneumoniae *	Peel	−	[27–29]
Oral			
*Staphylococcus aureus *	Peel	−	[30]
*Staphylococcus epidermidis *	Peel	−	[30]
*Streptococcus mutans *	Peel	−	[30, 31]
*Streptococcus salivarius *	Peel	−	[30]
*Streptococcus sanguis *	Peel	−	[31]
*Streptococcus mitis *	Peel	−	[31]
*Porphyromonas gingivalis *	Peel	−	[32, 33]
*Aggregatibacter actinomycetemcomitans *	Peel	−	[32]
*Prevotella intermedia *	Peel	−	[33]
*Proteus* spp.	Peel	−	[34]
Drug resistant			
Methicillin-resistant *Staphylococcus aureus *	Peel	−	[35–41]
*Acinetobacter baumannii *	Peel	−	[42]
*Helicobacter pylori *	Peel	−	[43]
